# Facilitators and Barriers to Self-Volume Management in Older Patients with Chronic Heart Failure and Multimorbidity: A Qualitative Study

**DOI:** 10.3390/healthcare13182353

**Published:** 2025-09-18

**Authors:** Xin Xu, Yu Chen, Jiaxin Zhou, Shuying Li, Xinyue Dong, Zhiyun Shen

**Affiliations:** 1 School of Nursing, Fudan University, Shanghai 200032, China; 23211170034@m.fudan.edu.cn (X.X.); 23211170048@m.fudan.edu.cn (J.Z.); 24211170047@m.fudan.edu.cn (S.L.); 2 Zhongshan Hospital, Fudan University, Shanghai 200032, China; 09401190069@fudan.edu.cn (X.D.); shen.zhiyun@zs-hospital.sh.cn (Z.S.)

**Keywords:** self-management, volume management, aged, chronic heart failure, multimorbidity, qualitative

## Abstract

Background: Effective volume management can significantly improve patients’ health outcomes, but the current situation of volume management in older patients with chronic heart failure (CHF) and multimorbidity is not optimistic. This study aimed to explore the facilitators and barriers of self-volume management in patients and to provide a basis for the development of self-volume management strategies. Methods: A descriptive qualitative research method was used. Semi-structured interviews were conducted with older patients with CHF and multimorbidity between January and April 2025 in two tertiary hospitals in Shanghai, China. Data were analyzed using content analysis. Results: Eight facilitators emerged, including the hospital–community collaboration mechanism, Medicare and long-term care insurance coverage, diverse social support, the doctor–patient trust relationship, results-oriented incentives, digital health management, high self-efficacy, and strong motivation for health. Nine barriers were identified; these were insufficient adaptability of self-volume management programs, limited access to community resources, lack of standardized self-volume management tools, inadequate multidisciplinary team communication, one-way doctor–patient communication, lack of knowledge of self-volume management, physical limitations, management negligence caused by work constraints, and behavioral habits’ consolidation. Conclusions: Self-volume management was affected by various factors. The study suggests strengthening health insurance coverage to reduce financial burden, taking advantage of family support and providing digital health management tools. In addition, healthcare providers should provide patient-centered care, enhance multidisciplinary collaboration, and address individual barriers with precise intervention strategies.

## 1. Introduction

Chronic heart failure (CHF) has become a significant challenge in global public health due to its high prevalence and readmission rates [1]. There are approximately 64.34 million HF patients worldwide [2], with around 6.7 million patients in the United States [3]. In China, a nation with a significant prevalence of cardiovascular disease, the HF population has exceeded 8.9 million, showing a continuous growth trend [4]. Notably, the incidence of CHF gradually increases with age, with a prevalence of 10.5% in people ≥65 years old and 20.0% in people ≥75 years old [5]. Due to the need for long-term medication maintenance and repeated hospitalizations, older patients with CHF not only face severe health threats but also impose a heavy economic burden on the global healthcare system [6].

The volume management, constructed based on self-management theory, facilitates the dynamic regulation of volume status, including patient-initiated fluid intake regulation, adherence to a sodium-restricted diet, and symptom monitoring [7]. Expert consensus indicates the importance of self-volume management [8]. Ezekowitz et al. implemented the Sodium-HF trial and found that patients in the intervention group who received a low-sodium diet with a target sodium intake of <1500 mg/d improved cardiac function and quality of life, as compared with the group who received a standard beverage [9]. A meta-analysis showed that sodium intake restriction in patients effectively improved quality of life and systolic blood pressure and lowered BNP or NT-proBNP levels [10].

Despite accumulated empirical evidence in volume management, a critical research gap remains in understanding the determinants of self-volume management among older patients with CHF and multimorbidity. The complexity of self-volume management in this population is of particular concern [11], as 58.7% of patients aged ≥65 years suffer from ≥5 chronic diseases, among which hypertension, diabetes mellitus, and chronic kidney disease substantially impact readmission rates and all-cause mortality [12]. Multimorbidity presents numerous clinical challenges, including but not limited to therapeutic conflicts associated with multiple medications, age-related physiologic decline, and conflicting disease management goals, which collectively constitute a complex self-management decision dilemma [13]. This phenomenon underscores the limitation of traditional standardized intervention models in effectively addressing the complex and evolving health needs of older adults with multimorbidity. Consequently, these patients face unique challenges in self-volume management.

Research has demonstrated the importance of self-volume management, but patient adherence remains suboptimal. Only 14% of patients report regular weight monitoring, and 9% engage in symptom monitoring [14]. Poor long-term adherence is primarily driven by inappropriate fluid intake, poor medication adherence, and insufficient symptom monitoring [14]. Nadrian et al. found significant variations in patients’ perceptions of self-volume management, which influenced their self-management behaviors and health outcomes [15]. While existing research on self-volume management is primarily quantitative, there is a limited in-depth qualitative exploration of patients’ experiences [9,10]. Lee et al. suggested that health status, social support, self-care, and socio-economic factors significantly influenced adherence to sodium-restricted diets [16]. Heo et al. identified barriers to management, including cognitive deficits, distorted perceptions, and insufficient family support, alongside facilitators such as dietary modification and positive family interactions [17]. Chinese patients, in particular, encounter contextual challenges such as insufficient skills, ageing mentalities, and inadequate social support systems [18]. Although previous studies have mainly explored the self-volume management experiences of elderly patients with HF, the specific barriers and facilitators for older patients with CHF and multimorbidity remain poorly understood.

Therefore, based on the Consolidated Framework for Implementation Research (CFIR) [19], this study systematically deconstructs the facilitators and barriers to self-volume management in older patients with CHF and multimorbidity. The results will help to develop a targeted intervention program designed to provide patients with continuous and practical support to enhance their self-volume management abilities and improve health outcomes.

## 2. Conceptual Framework

CFIR is the most widely used determinant analysis tool of the implementation process due to its multidimensional structural characteristics [20]. CFIR provides researchers with a structured approach through 48 constructs in five dimensions: innovation, outer setting, inner setting, individuals, and the implementation process [19,21], to facilitate multi-scenario, stage-by-stage analysis of influencing factors in intervention implementation.

The multidimensional features of CFIR make it particularly suitable for studying self-volume management. First, self-volume management involves multifaceted dimensions such as symptom monitoring, behavioral adjustment, and environmental adaptation. The complexity calls for a multi-level, multi-dimensional interactive evaluation framework. CFIR meets this need by enabling evaluation through specific constructs such as “adaptability” in the innovation domain, which assesses the individualized appropriateness of interventions, and “available resources” in the inner setting domain, which helps deconstruct the effectiveness of the support system [19,22]. Second, CFIR is highly applicable to research subjects. Previous studies have demonstrated its utility in the field of chronic diseases [23,24], with particular emphasis on its cultural adaptability and clinical relevance—especially in Chinese healthcare contexts through localized applications [25]. Additionally, CFIR facilitates the exploration of stakeholders’ perceptions and perspectives [26], which allows precise identification of facilitators and barriers to volume management. This capability provides a theoretical foundation for developing patient-centered precision intervention strategies.

Therefore, the purpose of applying CFIR is to explore the multidimensional determinants of self-volume management. Its use offers an innovative research paradigm for formulating tailored self-volume management strategies for older patients with CHF and multimorbidity.

## 3. Materials and Methods

### 3.1. Study Design

A descriptive qualitative study was performed to investigate facilitators and barriers to the application of self-volume management in older patients with CHF and multimorbidity. The study followed the Consolidated Standards for Reporting Qualitative Research (COREQ) and was conducted according to the Declaration of Helsinki [27] (see File S1). The study has been approved by the Ethics Committee of School of Nursing, Fudan University (IRB#2024-04-5).

### 3.2. Sample Selection and Recruitment

Purposive sampling was used to recruit interviewees for this study. Interviewees’ recruitment was conducted from January to April 2025 in the cardiology departments of two tertiary hospitals in Shanghai. Both hospitals are well known for providing skilled healthcare for older patients and the treatment of HF. With the highest rates of hospital admission for older patients in Shanghai, these two hospitals provide a clinically representative sample population for exploring the determinants of self-volume management. The research team evaluated potential interviewees through the hospital inpatient system to identify older patients with multimorbidity of diverse illness severity.

The study followed the principle of maximum differential sampling by selecting interviewees with significant differences in age, education, duration of CHF, and cardiac function classifications. The inclusion criteria were as follows: (1) age ≥ 60 years; (2) diagnosis of CHF following the guidelines for the diagnosis and treatment of heart failure; (3) New York Heart Association (NYHA) classification of II–IV; and (4) presence of one or more comorbidities. Exclusion criteria were those individuals with mental disorders and impaired cognition. All interviewees were recruited directly by members of the research team. The sample size was determined based on data saturation. Recruitment was terminated when no new codes or new dimensions appeared in three consecutive interviews [28]. The purpose, risks, and benefits of the study, as well as the principles of confidentiality and voluntary participation, were explained to the interviewees either verbally or by text.

### 3.3. Data Collection

The interview outline (File S2) was developed based on the study’s objectives and the CFIR, following a review of the relevant literature and discussion in the research team. Two older patients with CHF and multimorbidity were selected for pre-interviews. The results showed that the interview questions were clear and effective in guiding the expression of the topic, so the outline was not revised. The data collection was independently completed by the first researcher (a female master’s degree student in nursing who completed 2 years of training in qualitative research methodology). The research process was supervised by the second author (associate professor of chronic disease nursing with extensive experience in interviewing and surveying), including research activities and data collection procedures. The researcher maintained continuous communication with the interviewees through the preclinical practicum and established a trusting relationship, strictly following the “data collector–clinical caregiver” role separation principle.

Data were collected through one-on-one, face-to-face, and semi-structured interviews, without the involvement of a third party. The interviews were held in a quiet, private, and undisturbed setting in the doctor’s on-call room. Both interviewer and interviewees conducted the interviews in Mandarin Chinese. This enabled them to clearly and accurately share their experiences and feelings during the interviews. With the consent of the interviewees, the entire conversation was audio-recorded with a tape recorder to ensure the integrity of the interview materials, and the researcher managed the recording and interview materials confidentially. To encourage the interviewees to freely and fully express their inner feelings and experiences, the interview began with the question, “How are you feeling now? Can you talk to me about your experience with the disease?” as an entry point to establish a relaxed and friendly interview atmosphere. During the interviews, close attention was paid to non-verbal information such as facial expressions, body language, and tone of voice. Continuous reflection was undertaken, and memos were written during the interviews to document emerging insights. Each interview lasted 30–60 min, and data collection and analysis were conducted simultaneously. Data collection was concluded following the study of 19 interviews since no new themes emerged and data saturation was reached [28].

### 3.4. Data Analysis

The researcher transcribed the interview contents within 24 h post-interviews and refined the information by combining the memos recorded during the interviews. The researchers returned the results to the interviewees for validation of completeness and accuracy. The interviewees were numbered based on the sequence of the interviews (interviewees: P1, P2, P3,…, P19). The study was systematically coded and classified by content analysis [29] and utilized the qualitative analysis software Nvivo 15.0 for data management. The steps were as follows: (1) according to the coding manual provided on the CFIR website, the definitions and illustrations of dimensions and constructs under the CFIR were clarified. (2) Interview data were read repeatedly for immersion in the data, and the overall contents of the data were grasped, including language and nonverbal expressions such as sighs, frowns, and tears. (3) The data were analyzed word by word, highlighting essential statements about self-volume management for open coding. (4) Similar or related codes against the categories in the CFIR coding manual were compared and categorized to develop themes gradually. (5) Adjustments were made to the coding themes during the analysis process, such as deleting codes not mentioned in the interview results and making appropriate adjustments to the code statements based on the data. One researcher conducted the data analysis, and a second researcher comprehensively reviewed the results. Any discrepancies were recorded, discussed, and resolved through consensus among all researchers.

To ensure the accuracy of data analysis and the fidelity of translation, the following step-by-step process was adopted. First, raw data processing and coding were conducted in a Chinese-language environment. This approach was taken to completely avoid any semantic loss or bias that might arise from translation during the core phase of data analysis. This ensured that the generated codes and themes were rooted in the original linguistic and cultural context. Second, presentation and translation of the research results (Chinese to English) were performed after finalizing the themes. This process was expertly managed by researchers within the team who possess professional proficiency in both Chinese and English. Third, to maximize translation quality, a multi-stage verification process was implemented. This involved human-led translation with the aforementioned researchers completing the initial translation, followed by technology-assisted verification using AI-assisted translation tools to polish the language.

## 4. Results

### 4.1. Demographic Characteristics of Interviewees

Twenty interviewees were recruited, with one declining due to privacy concerns. Ultimately, 19 interviewees were interviewed, and all provided signed informed consent. The interviewees ranged in age from 60 to 85 years, and comprised 13 males and 6 females. Nine interviewees had a senior high school education or higher, while 14 interviewees were retired. The disease duration spanned 1 to 30 years, and comorbidities consisted of 1 to 3 diseases. The demographic characteristics of the interviewees are shown in Table 1.

### 4.2. Facilitators of Self-Volume Management

Using content analysis, this study identified a total of 133 codes related to factors promoting self-volume management. Based on the five domains of the CFIR, semantically similar codes were grouped into 8 categories, which included 17 subcategories. These categories are the hospital–community collaboration mechanism, Medicare and long-term care insurance coverage, the doctor–patient trust relationships, results-oriented incentives, diverse social support, digital health information, high self-efficacy, and strong motivation for health, as detailed in Table 2.

#### 4.2.1. Hospital–Community Collaboration Mechanism

Most interviewees confirmed that a hospital–community collaboration mechanism had been constructed in their regions. This model allows patients to obtain professional guidance through systematic health education resource allocation (including health manuals and knowledge lectures). Interviewees reported that the hospital–community collaboration mechanism enhanced convenience and their ability to manage their volume.

*“The hospital and the community have a long-term cooperative relationship. It (the hospital) regularly organizes free medical consultations and lectures.”*

*“Salt-reduction spoons were distributed in the community, advising us to reduce our salt intake.”*

Some interviewees indicated that it was difficult for older adults to access online hospital services, while the community family doctor’s registration system solved the problem.

*“My family doctor is very responsible. He regularly asks about my conditions and makes appointments for me online.”*

#### 4.2.2. Medicare and Long-Term Care Insurance Coverage

Interviewees emphasized the crucial role of the health insurance system in alleviating financial strain. They mentioned that the reimbursement rate for hospitalization costs under local urban employee health insurance could reach 80%. Notably, interviewees highly regarded the pioneering long-term care insurance system in Shanghai, indicating they received subsidized care costs and home care services upon successful application.

*“I depend significantly on Medicare, which reimburses a substantial portion of my expenses.”*

*“I applied for long-term care insurance, which provides volunteers to assist with household tasks. There is also a monthly subsidy, which improved the quality of life.”*

#### 4.2.3. Diverse Social Support

The interviewees reported that, besides the help of healthcare providers, the companionship and support of family and friends were essential in self-volume management. Most interviewees indicated that they lived with their spouses or children. Support from their families helps them remain optimistic in the face of illness. In daily life, family members and friends reminded interviewees of diet, medication, and regular follow-ups, which increased interviewees’ motivation for self-volume management.

*“Since I became ill, my spouse has encouraged me to manage my condition and take my medication regularly.”*

*“My children show filial piety by providing care, accompanying me to the hospital for regular follow-up and medication adjustments.”*

*“When I go out to eat with my friends, they worry about my illness and advise me to eat less oily and salty food.”*

The interviewees indicated that support from patient peers effectively promoted and sustained their self-volume management, serving as a facilitator.

*“Patient peers who access the volume management information on their mobile devices often share it to exchange insights.”*

#### 4.2.4. Doctor–Patient Trust Relationship

Trust was regarded as a collective value or normative behavior within healthcare settings. Interviewees consistently identified “trust” as a core element of the doctor–patient relationship. The established trust directly translated into improved treatment adherence, thereby enhancing self-volume management compliance. In addition, interviewees reported that the doctors and nurses delivered commendable service, which made them more inclined to discuss their concerns and seek assistance.

*“The first thing I do when I’m sick is to go to the hospital to see a doctor. I feel that doctors at high-level hospitals are more professional and trustworthy.”*

*“All of the healthcare providers are great communicators, and we have mutual respect with them. I am also willing to follow their guidance.”*

#### 4.2.5. Results-Oriented Incentives

Interviewees indicated that visualized data feedback served as a powerful tool for self-monitoring, facilitating the observation of improvements in their physiological markers and overall physical conditions. These improvements fostered a more optimistic prognosis and, in turn, significantly boosted their motivation to adhere to daily symptom monitoring, regular weighing, and timely medications.

*“I reached 400 m in my 6-min walk test and experienced an improvement in my condition.”*

*“The physician reviewed the checklist and reported the enhancement in left ventricular ejection fraction, which means the management remains effective. I am willing to continue with it.”*

Some interviewees showed a keen interest in other patients’ successful disease management experiences. They perceived cases with similar experiences and better prognoses as significant motivators for their own management efforts.

*“Unless there is a heart failure patient who is older and in better health than me, then he serves as my role model.”*

#### 4.2.6. Digital Health Management

Interviewees reported that age and physical limitations, such as difficulty reading small font sizes and the effort required for prolonged reading, might impede their ability to read paper-based educational materials. Consequently, they preferred a digital health platform for information acquisition. The platform’s absence of temporal and spatial constraints, combined with its capacity for timely information updates, enhanced its convenience and applicability, making it more responsive to their needs.

*“I typically don’t read the brochures since I have presbyopia and my brain slows down. It is more convenient to use WeChat and TikTok.”*

*“The platform disseminates science videos that captivate my interest. I listen to them at night before retiring for sleep.”*

Some interviewees who received cross-regional medical treatments preferred the services offered by internet hospitals due to considerations of distance and time expenditure.

*“I live in another province, far away from a tertiary hospital. I chose to seek medical treatment at an internet hospital. ”*

Interviewees utilized smart bracelets to monitor physiological indicators like heart rate, blood pressure, and sleep, thereby facilitating effective health management.

*“This fitness wristband is beneficial since it transmits real-time health data to my phone, assists in symptom monitoring.”*

#### 4.2.7. High Self-Efficacy

Self-efficacy denotes an individual’s confidence in managing a disease and performing health-related tasks effectively [30]. Interviewees in this study attained significant self-efficacy in long-term disease management, leading to enhanced confidence and sustained adherence.

*“I’m feeling good about things and I’m just going to stick to what the doctor says.”*

*“Stay positive. I’m feeling more in control now compared to before.”*

#### 4.2.8. Strong Motivation for Health

Health motivation, defined as the enduring driving force system formed by patients through intrinsic value assessments during disease management, is the fundamental mechanism for behavior change [31]. This motivation system encourages active engagement in volume management and timely medication adherence. Interviewees reported that health motivation resulted from the importance of personal health, awareness of disease consequences, and the desire to enhance quality of life.

*“It is essential to advocate for involvement in volume management. Ultimately, the well-being of the body is what matters, unless you don’t want to live.”*

*“Anything beneficial to the body is eager to implement it.”*

### 4.3. Barriers to Self-Volume Management

This study identified a total of 63 codes related to factors hindering self-volume management. It included 9 categories and 17 subcategories. These categories include insufficient adaptability of management programs, limited access to community resources, lack of standardized volume management tools, inadequate multidisciplinary team communication, one-way doctor–patient communication, lack of knowledge of self-volume management, physical limitations, management negligence caused by work constraints, and behavioral habits’ consolidation, as detailed in Table 3.

#### 4.3.1. Insufficient Adaptability of Self-Volume Management Programs

Most interviewees reported considerable differences in their individual needs for self-volume management programs, as older patients had comorbidities. They believed that effective management should focus on patients. However, the current programs lacked flexibility and applicability.

*“For someone with diabetes and kidney illness like myself, some of the advice is inappropriate.”*

*“Since every situation is unique and there is no one-size-fits-all solution, management must consider the patient’s actual circumstances (frown).”*

#### 4.3.2. Limited Access to Community Resources

The multifaceted services provided by the community, such as health education, life care, and medical support, play an essential role in promoting the sustainability of self-volume management. However, some interviewees indicated that the lack of resources might affect their self-volume management.

*“The community’s capacity is limited, and funding is not available every year.”*

*“There are no lectures offered in the community for heart failure disease, so I don’t find the lectures very helpful.”*

*“The medicine prescribed by the community hospital is insufficient for my needs.”*

#### 4.3.3. Lack of Standardized Self-Volume Management Tools

Standardized volume management instruments can efficiently assist patients in self-volume management. However, interviewees reported challenges in executing management strategies owing to the lack of such standardized tools. Moreover, the scarcity of usage instructions for available volume management tools potentially led to patients’ inadequate proficiency in their application.

*“The only management tool I am aware of is the scale, and I am not familiar with any others.”*

*“When communities distribute salt-control spoons and oil-control bottles, it’s best to include instructions on exactly how to use them.”*

#### 4.3.4. Inadequate Multidisciplinary Team Communication

The volume management of older patients with CHF and multimorbidity presents clinical complexity. Some interviewees reported confusion and concerns about comorbidity management and medication use due to inadequate communication between multidisciplinary teams. Furthermore, they highlighted the absence of consensus on prioritizing therapeutic goals when managing comorbidities that conflicted with volume management, leading to significant distress.

*“You see, I have gout, and I have to drink a lot of water. But I should restrict my water intake due to heart failure.”*

*“My medication for high blood pressure may cause hyperkalemia, and I’m concerned about whether this will affect my heart.”*

#### 4.3.5. One-Way Doctor–Patient Communication

Most interviewees reported insufficient communication and interaction with their physicians. This one-way doctor–patient communication was evident in the predominant role of health care in formulating management programs and health guidance. Patients’ feedback and needs were insufficiently acknowledged or addressed.

*“We are simply following the doctor’s advice.”*

*“The doctor was too busy and didn’t have much time to talk with us.”*

#### 4.3.6. Lack of Knowledge of Self-Volume Management

Interviewees were affected by a lack of knowledge and struggled to manage their self-volume well in a multimorbid state. The symptom monitoring, medication management, and volume management primarily relied on subjective experiences, without specific and clear guidance, which impeded interviewees’ self-management behaviors.

*“I am not sure if diabetic diets and heart failure diets conflict with each other.”*

*“When I feel better, I automatically reduce and stop my medication. I don’t know that some medications cannot be stopped.”*

#### 4.3.7. Physical Limitations

Interviewees reported diminished self-volume management and a lower quality of life, largely due to physical function limitations that hindered their ability to implement self-volume management. Furthermore, interviewees indicated that they were aging, and this aging mindset fostered negative beliefs, which also presented a barrier to self-volume management.

*“The quality of life affected by the disease is getting worse and worse. As I get older, it seems like all the efforts I make don’t have a significant effect.”*

*“Memory loss often causes me to forget to take my medication.”*

*“I’m too old to need guidance now, so I just get by.”*

#### 4.3.8. Management Negligence Caused by Work Constraints

Some interviewees indicated that the low priority given to self-volume management was mainly due to work pressure. They reported that their busy work often left them with insufficient time to dedicate to self-volume management.

*“There is just not enough time during the week because of the busy work.”*

*“As soon as I get into work, I can’t remember to take my medication, and sometimes I forget to manage it.”*

#### 4.3.9. Behavioral Habits’ Consolidation

Behavioral habits’ consolidation, referring to the long-standing patterns of behavior such as eating and lifestyle habits that interviewees develop over many years, significantly influences self-volume management. Interviewees regarded these ingrained habits as obstacles to effective management, especially when they conflicted with current disease management protocols.

*“In my previous job, I ran long distances and drank several bottles of green tea a day, and it was hard to break the habit.”*

*“I used to enjoy pickles and salted foods. It’s now difficult to avoid eating salty foods.”*

## 5. Discussion

This study focused on facilitators and barriers to implementing self-volume management in older patients with CHF and multimorbidity, using qualitative research to capture patients’ experiences and perceptions. CFIR-based systematic coding revealed that the influences on patients’ self-volume management could be categorized into eight facilitators and nine barriers, covering multiple dimensions including innovation, outer setting, inner setting, individuals, and implementation process. These factors indicated the complexity of self-volume management in older patients with CHF and multimorbidity.

### 5.1. Facilitators: The Dual Role of System Support and Individual Agency

The facilitators identified in this study are predominantly at the system level, aligning with global initiatives aimed at enhancing chronic disease management. Collaboration between hospitals and communities is crucial for effective healthcare delivery, which embodies the integrated, patient-centered care model endorsed by the World Health Organization [32]. Evidence from various countries supports this. For instance, in the United States, improved communication and collaboration within the healthcare system have demonstrated positive impacts on community health outcomes and health equity [33]. In Australia, chronic disease management prioritizes coordination between hospitals and communities, which reduces readmissions by ensuring continuity and integration of care services [34]. In Japan, healthcare providers emphasize the importance of information sharing through inter-institutional collaboration [35]. Given this evidence, it is recommended that a unified electronic health record (EHR) platform be established to enable the secure and authorized sharing of patient information between hospitals and communities, thereby removing information barriers. Carter et al. indicated that integrating digital platforms with community health providers for the remote monitoring of patients with CHF was both feasible and well-accepted [36]. This supports further research on mobile health platforms. Additionally, this study demonstrated that hospital–community collaboration not only facilitated structural integration but also underpinned the development of strong trust between healthcare providers and patients. Trust is a critical influence on treatment adherence and self-volume management behavior [37].

Interviewees frequently identified health insurance and long-term care insurance coverage as significant factors. This finding is consistent with international research on healthcare accessibility and its influence on disease burden [38]. In high-income countries with universal health insurance coverage, improved quality and accessibility of medical services have led to greater adherence to treatment plans [38,39]. For example, the Australian government has reduced readmission rates and enhanced patient self-management through investments in the Hospitalization Risk Plan [34]. In contrast, patients in low-income countries often face higher out-of-pocket medical expenses [40]. Those countries continue to encounter barriers to universal health coverage, particularly regarding equitable access for elderly and low-income populations [41,42]. The present findings indicate that reducing the economic burden is fundamental to effective self-management interventions, especially for older people with multiple comorbidities, frequent medical visits, and polypharmacy. Future policies should link health insurance payments to quality indicators, such as readmission rates, patient-reported outcomes, and dry weight achievement rates. This approach would move beyond traditional fee-for-service models by encouraging healthcare institutions to prioritize preventive and management services. Additionally, improving the quality of insurance services for vulnerable elderly populations is crucial for addressing existing health equity challenges.

At the individual level, high self-efficacy and strong health motivation represent intrinsic motivation, aligning with Bandura’s social cognitive theory [43]. This study enriches the theory by providing qualitative evidence that self-efficacy in elderly patients with comorbidities was reflected in family support and outcome-oriented incentive mechanisms. The study identified that family support influenced self-volume management efficacy through a dual role. First, it acts as an external support system through behavioral monitoring, with family members providing regular medication reminders, follow-up appointments, and health behavior supervisions. Secondly, family support contributes to psychological empowerment, enhancing patients’ self-efficacy and sustaining management of self-volume through emotional support, which aligns with the findings of Doris et al. [44]. The familistic culture in Chinese society offers a distinct advantage. Collectivist values enhance the sense of responsibility among family members, thereby facilitating patients’ acceptance of health behavior monitoring. For future practice, it may be worthwhile to consider integrating family members as care partners. This could involve actively inviting them to participate in educational meetings and discharge planning, and training them to master key capacity management skills, such as daily weight measurement, checking for lower limb edema, and recognizing worsening heart failure signs. Additionally, it is essential to build outcome-oriented incentive mechanisms, such as visual feedback and sharing of success stories, to enhance patients’ perception of benefits and ultimately promote long-term management behaviors.

Digital health management tools represent a promising means of enhancing healthcare delivery. These technologies improve patient access to and management of health information by enabling real-time data transmission, remote monitoring of physiological parameters, and automated medication reminders. Similar outcomes have been reported in the United Kingdom [45], Spain [46], and the United States [47]. Leveraging digital technology can further expand the accessibility and effectiveness of health education. Consequently, reliable mobile health applications should be identified or developed to facilitate patient self-management, including recording weight, tracking symptoms, monitoring medication adherence, and setting automated reminders and alerts. Addressing the digital divide remains essential. Targeted training for older adults and provision of non-digital alternatives are necessary to ensure equitable access to health management resources.

### 5.2. Barriers: Revealing the Gap Between Guidelines and Practice

The barriers identified in this study profoundly revealed the “last mile” challenge of translating evidence-based guidelines into daily practice. The lack of adaptability and standardized tools in self-volume management plans reflects intervention complexity. Effective self-volume management must accommodate diverse patient needs, encompassing endogenous factors, such as age-related cognitive decline, conflicting treatment goals from multiple comorbidities, and varying health literacy levels, as well as exogenous factors, such as the adequacy of social support systems and the economic burden of medications. These differences influenced patients’ self-volume management behaviors and adherence, aligning with Tayebe et al. [48]. Aggarwal et al. proposed that management strategies should be individualized and dynamically adjusted for complex situations involving multiple medications and comorbidities [49]. International examples also offer valuable insights. Skou et al. in Denmark improved the quality of life of patients with comorbidities through personalized self-management support [50], and a European customized care plan platform offered decision support and empowerment [51]. Additionally, the lack of standardized tools hindered management. Most interviewees lack a systematic understanding of volume management tools, leading to low efficiency and a breakdown in the scientifically standardized management loop [52]. Currie et al. noted that even with educational manuals, patients felt ill-informed due to the absence of specific guidance [53]. Given the complexity of comorbidities, establishing the role of HF case managers to develop individualized self-volume management plans and provide tools and guidance is a worthwhile consideration.

In relation to the communication aspect, inadequate multidisciplinary team communication and one-way doctor–patient communication represent significant barriers to effective care. Interviewees reported that age and comorbidities exacerbated communication deficiencies, resulting in conflicts regarding polypharmacy and treatment prioritization. One-way doctor–patient communication contradicted patients’ expectations of a “patient-centered” bilateral communication model, which negatively impacted treatment adherence. While guidelines recommend multidisciplinary teams, their implementation is key [54]. Examples like Grady Health System’s interdisciplinary collaboration in the United States [55] and Europe’s care planning platform [51] demonstrated improved patient-centered care and decision-making. Meta-analyses further confirmed that multidisciplinary approaches reduced hospitalizations, improved quality of life, and decreased mortality [56]. Therefore, future strategies should focus on the establishment of structured, interdisciplinary teams and the use of standardized communication tools to strengthen patient empowerment and deliver patient-centered care.

Regarding the individual aspect, barriers are multifaceted and intertwined. The interviewee reported relying on subjective experience due to a lack of systematic knowledge and guidance. Self-management expertise is not just about information, but how it is conveyed, interpreted, and internalized [57], necessitating patient-centered education with feedback methods for enhanced capacity. These feedback methods have shown proven effectiveness in the Republic of Korea [58], Iran [59], and Vietnam [60]. Secondly, management neglect caused by work pressure highlighted the conflict between social roles and health management. Despite being elderly and from low-income families in China, some patients continued working or farming to support themselves. This work-related management opportunity gap means that while patients are aware of the importance of management, they struggle to put it into practice, creating a “know but cannot act” scenario.

Additionally, physical limitations and behavioral habits’ consolidation, stemming from physiological and long-term patterns, present challenges. Elderly patients often experience physical decline accompanied by vision impairments and cognitive decline, making it challenging to monitor weight and record medication use. Furthermore, long-established dietary habits frequently conflict with management behaviors. Watson et al. suggested that nutritional habits were unconscious biological behavioral patterns reinforced through repetition, thereby strengthening the intensity of the habit [61]. This challenge arises from patients’ unconscious reactions rooted in established habits, creating psychological dependence. Simultaneously, changing established habits may trigger negative emotions such as anxiety and resistance, weakening the willingness to act [62]. It is recommended that clinical practitioners use habit replacement therapy as an intervention, employing progressive behavioral restructuring strategies to help patients establish new, adaptive behavioral patterns, thereby reducing psychological resistance to habit change.

### 5.3. Implications for Policy and Practice

The results of this study suggest the need to advance management implementation across three key dimensions—systemic, individual, and policy. Comprehensive efforts should be made to enhance the accessibility and equity of health services.

At the systemic level, hospital–community collaboration mechanisms can be established. Shared digital health platforms should be leveraged to break down data silos, enabling dynamic monitoring and early warning of patient volume management indicators. In addition, diverse social support resources should be integrated to build multidimensional support networks. At the individual level, patient-centered personalized management strategies are advocated for, emphasizing shared decision-making between physicians and patients. Leveraging the strengths of Chinese family culture, family-participatory management plans could be implemented to enhance patient adherence and self-efficacy. At the policy level, policymakers should strive to improve medical insurance and economic support systems. Addressing the complex healthcare needs of older patients with multimorbidity, it is suggested to expand coverage and optimize reimbursement catalogs to reduce financial burdens, so as to eliminate health inequalities. Additionally, it is encouraged to develop and promote age-friendly digital health products and services, empowering older patients to actively participate in self-volume management.

### 5.4. Strengths and Limitations

The study is one of the few qualitative studies on self-volume management among older patients with CHF and multimorbidity in China. It focused on the specific health challenges faced by this population in the Chinese context. This study systematically explored the determinants of self-volume management. It aims to address the complex clinical challenges associated with physiological decline, polypharmacy, and limited volume management capabilities that are characteristic of this group. This study innovatively used the CFIR as a theoretical framework and systematically deconstructed the facilitators and barriers under five constructs. These findings enhance the understanding of self-volume management in older patients in China and establish a foundation for developing targeted intervention strategies in the future.

However, this study has certain limitations. The data were collected from two tertiary hospitals, and the sampling did not include primary care and outpatient groups. As a result, there may be selection bias due to differences in healthcare resource allocation, which limits the generalizability of the study’s findings to other healthcare settings. Additionally, this study used semi-structured interviews for data collection. Still, it did not include ongoing, dynamic monitoring of patients’ self-volume management, nor did it feature a longitudinal analysis of how these behaviors may change over time.

## 6. Conclusions

This research provides valuable insights into the facilitators and barriers to self-volume management among older patients with CHF and multimorbidity. Eight facilitators were identified: the hospital–community collaboration mechanism, Medicare and long-term care insurance coverage, the doctor–patient trust relationship, results-oriented incentives, diverse social support, digital health management, high self-efficacy, and strong motivation for health. Notably, the research highlights the crucial role of the health insurance system in significantly reducing patients’ financial burdens, indicating that policymakers should focus on addressing patient needs and enhancing insurance coverage for this population. Additionally, the study emphasized the importance of family support within the Chinese socio-cultural context, underscoring the need to reinforce this support system.

On the other hand, several barriers hindered effective self-volume management, including limited access to community resources, inadequate multidisciplinary team communication, one-way doctor–patient communication, and a lack of standardized self-volume management tools. These findings pointed to the necessity of adopting a patient-centered care model and enhancing multidisciplinary collaboration. Barriers in individual domains, such as the lack of knowledge of self-volume management, physical limitations, management negligence caused by work constraints, and behavioral habits’ consolidation, further complicate effective self-volume management. Addressing these individual challenges through targeted intervention strategies is essential for improving self-volume management outcomes.

## Figures and Tables

**Table 1:** Description of study interviewees (*n* = 19).

ID	Sex	Age (years)	Education Level	Employment	Disease Duration (years)	NYHA Classification	Multimorbidity
P1	F	77	Junior High School	Retirement	30	Ⅱ	Hypertension, Diabetes, COPD
P2	M	66	Elementary School	Peasant	2	Ⅱ	APE, Pericardial Effusion
P3	M	72	Junior College	Retirement	5	Ⅱ	Gout
P4	M	75	Junior High School	Retirement	8	Ⅱ	Diabetes, CRI
P5	M	64	Senior High School	Retirement	5	Ⅲ	Hypertension, Diabetes, Renal Artery Stenosis
P6	F	81	Junior High School	Retirement	4	Ⅲ	Hypertension, Diabetes, CRI
P7	F	80	Senior High School	Retirement	2	Ⅱ	Diabetes
P8	M	65	Junior High School	Retirement	1	Ⅱ	Diabetes
P9	F	72	Senior High School	Retirement	17	Ⅲ	Diabetes, CRI
P10	M	63	Senior High School	Public Servant	2	Ⅱ	Hypertension
P11	F	83	Junior High School	Retirement	6	Ⅲ	Hypertension, Diabetes
P12	M	60	Junior High School	Freelance Work	5	Ⅱ	Hypertension
P13	F	85	Senior High School	Retirement	3	Ⅲ	Hypertension, Diabetes, CRI
P14	M	69	Senior High School	Retirement	2	Ⅱ	CRI
P15	M	64	Junior High School	Retirement	4	Ⅳ	Diabetes
P16	M	79	Junior High School	Retirement	1	Ⅱ	Hypertension, Dermatomyositis
P17	M	67	Undergraduate	Community Manager	2	Ⅲ	Hypertension, Diabetes, CRI
P18	M	60	Junior High School	Worker	5	Ⅳ	Hypertension
P19	M	69	Senior High School	Retirement	7	Ⅲ	Diabetes

F = female, M = male. COPD = chronic obstructive pulmonary disease, APE = acute pulmonary embolism, CRI = chronic renal insufficiency, NYHA = New York Heart Association.

**Table 2:** Facilitators of self-volume management in older patients with CHF and multimorbidity.

Domain	Categories	Subcategories	Statements	Number of Codes	Total Number of Codes
Outer Setting	Hospital–community collaboration mechanism	Community hospital partnership	The hospital and my community maintain a strong partnership with a team of specialized physicians who provide health seminars across the neighborhoods. I have been paying attention to these activities since my diagnosis of heart failure.	6	11
		Community outreach and education	The community hands out a citizen’s health handbook and a salt-control spoon, and they recommend adding a teaspoon of salt to regulate salt consumption.	3	
		Sign up for a family doctor	The family doctor is attentive to my condition and discusses the treatment plan with me. He assists me in scheduling an appointment with a highly regarded expert at the hospital, which is helpful since I cannot manage all of this independently.	2	
	Medicare and long-term care insurance coverage	Medical insurance	Paying for medical treatment with medical insurance significantly reduced my expenses. Medical insurance covers over 80% of the costs, which is still very good.	13	17
		Long-term care insurance	Long-term care insurance provides volunteers to assist with household tasks. There is also a monthly subsidy, which has improved the quality of life.	4	
	Diverse social support	Ongoing spousal companionship	Since I got sick, my partner has consistently supported me by encouraging adherence to management strategies and reminding me to take my medication.	15	44
		Support from family and nanny	My children show filial piety by providing care, accompanying me to the hospital for regular follow-up and medication adjustments. The nanny is more conscious of the type of food I choose to eat because of my illness and helps me heat my herbs every day.	15	
		Thoughtful reminders and support from friends	My friends are concerned about my condition and have advised me to eat less oily and salty food. Patient peers who access the volume management information online will share it.	14	
Inner Setting	Doctor–patient trust relationship	Trust healthcare providers	Doctors at large hospitals carry more credibility, so I tend to seek their advice. Due to my trust in these physicians, I consistently adhere to their recommended management strategies.	6	10
		Harmonious relationship	All of the healthcare providers are great communicators, and we have mutual respect for them. I am also willing to follow their guidance.	4	
	Results-oriented incentives	Incentives for quantitative indicators	I reached 400 m in my 6 min walk test and experienced an improvement in my condition. The physician reviewed the checklist and reported the enhancement in left ventricular ejection fraction, which means the management remains effective.	5	11
		Motivation from successful cases	If there are patients older than me who are in better health, they serve as role models for me. I am more willing to learn from them.	6	
	Digital health management	Online education	I typically don’t read the brochures since I have presbyopia and my brain slows down. It is more convenient to use WeChat and TikTok.	18	22
		Effective data monitoring	This fitness wristband is beneficial since it transmits real-time health data to my phone, alerts me of outliers, and assists in symptom monitoring. I review these data when possible.	4	
Individuals	High self-efficacy	Positive attitude	I’m feeling good about things, so I’m just going to stick to the doctor’s advice. Stay positive. I’m feeling more in control now compared to before.	6	6
	Strong motivation for health	Cherish life	It is essential to advocate for involvement in volume management. Ultimately, the well-being of the body is what matters, unless you don’t want to live.	8	12
		Pay attention to diseases	I’m happy to follow any management advice that benefits my health.	4	
Total	8 categories	17 subcategories			133

**Table 3:** Barriers to self-volume management in older patients with CHF and multimorbidity.

Domain	Categories	Subcategories	Statements	Number of Codes	Total Number of Codes
Innovation	Insufficient adaptability of management programs	Complex needs of comorbid conditions	For someone with diabetes and kidney illness like myself, some of the advice is inappropriate.	5	7
		Practical needs of the programs	Individual physical conditions vary significantly; therefore, standardized recommendations may not be appropriate for all patients. Management must consider the patient’s actual circumstances.	2	
Outer Setting	Limited access to community resources	Limited funds	The community’s capacity is also limited, and funding is not available.	5	9
		Limited medical resources	The medicine prescribed by the community hospital is insufficient for my needs.	4	
	Lack of standardized volume management tools	Lack of toolkits	The only management tool I am aware of is the scale, and I am not familiar with any others.	5	8
		Lack of supporting guidance	When communities distribute salt-control spoons and oil-control bottles, it’s best to include instructions on exactly how to use them.	3	
Inner Setting	Inadequate multidisciplinary team communication	Concerns about polypharmacy	My medication for high blood pressure may cause hyperkalemia, and I’m concerned about whether this will affect my heart.	4	5
		The trouble of comorbidities	I have gout, and I have to drink a lot of water. But I should restrict my water intake due to heart failure.	1	
	One-way doctor–patient communication	Unidirectional compliance	We are simply following the doctor’s advice.	6	8
		Lack of communication	The doctor was too busy to talk to us.	2	
Individuals	Lack of knowledge of self-volume management	Lack of medication knowledge	When I feel better, I automatically reduce and stop my medication. I don’t know that some medicines cannot be stopped.	8	11
		Lack of knowledge about comorbidity management	I have proteinuria and am unsure about my diet. I am not sure if diabetic diets and heart failure diets conflict with each other.	3	
	Physical limitations	Physical decline	As I get older, it seems that all the efforts I make have little to no significant effect.	3	7
		Memory impairment	Memory loss often causes me to forget to take my medication.	2	
		Mental aging	I’m too old to need guidance now, so I manage just fine.	2	
	Management negligence caused by work constraints	Lack of management opportunities	There isn’t enough time during the week due to the busy work. As soon as I get into work, I can’t remember to take my medication, and sometimes I forget to manage it.	2	2
	Behavioral habits’ consolidation	Inherent lifestyle habits	I used to eat pickled vegetables, but now I find it challenging to change my eating habits.	5	6
		Inherent professional habits	In my previous job, I ran long distances and drank several bottles of green tea a day, which made it hard to break the habit.	1	
Total	9 categories	18 subcategories			63

## Data Availability

The dataset is available on reasonable request from the corresponding author due to ethical restrictions.

## Supplementary Materials

The following supporting information can be downloaded at: https://www.mdpi.com/article/10.3390/healthcare13182353/s1, File S1: Qualitative research reporting synthesis; File S2: Interview outline.
