# Supplementary material for: Facilitators and Barriers to Self-Volume Management in Older Patients with Chronic Heart Failure and Multimorbidity: A Qualitative Study
Source: Healthcare (Basel). 2025 Sep 18;13(18):2353. doi: 10.3390/healthcare13182353 (PMC12469624; doi:10.3390/healthcare13182353)
Supplement: Supplementary file 1 [file healthcare-13-02353-s001.zip › File S1 Qualitative reaearch reporting.pdf]

Consolidated criteria for reporting qualitative studies (COREQ):

32-item checklist

Developed from:

Tong A, Sainsbury P, Craig J. Consolidated criteria for reporting qualitative research (COREQ): a 32-item checklist for interviews and focus groups. Int J Qual Health Care.2007 Dec;19(6):349-57.

No. Item	Guide questions/description	Reported on Page #
Domain 1: Research team and reflexivity		
Personal Characteristics		
1. Interviewer/facilitator	Which authors conducted the interview or focus group?	4
2. Credentials	What were the researcher's credentials? E.g. PhD, MD	4
3. Occupation	What was their occupation at the time of the study?	4
4. Gender	Was the researcher male or female?	4
5. Experience and training	What experience or training did the researcher have?	4
Relationship with participants		
6. Relationship established	Was a relationship established prior to study commencement?	4
7. Participant knowledge of the interviewer	What did the participants know about the researcher? *e.g. personal goals, reasons for doing the research*	4
8. Interviewer characteristics	What characteristics were reported about the interviewer/facilitator? e.g. Bias, assumptions, reasons and interests in the research topic	4
Domain 2: study design		
Theoretical framework		
9. Methodological orientation and Theory	What methodological orientation was stated to underpin the study? e.g. grounded theory, discourse analysis, ethnography, phenomenology, content analysis	4
Participant selection		
10. Sampling	How were participants selected? e.g.	4

	purposive, convenience, consecutive, snowball	
11. Method of approach	How were participants approached? e.g. face-to-face, telephone, mail, email	4
12. Sample size	How many participants were in the study?	5
13. Non-participation	How many people refused to participate or dropped out? Reasons?	4
Setting		
14. Setting of data collection	Where was the data collected? e.g. home, clinic, workplace	4
15.Presence of nonparticipants	Was anyone else present besides the participants and researchers?	4
16. Description of sample	What are the important characteristics of the sample? e.g. demographic data, date	4-5
Data collection		
17. Interview guide	Were questions, prompts, guides provided by the authors? Was it pilot tested?	4
18. Repeat interviews	Were repeat interviews carried out? If yes, how many?	4
19. Audio/visual recording	Did the research use audio or visual recording to collect the data?	4
20. Field notes	Were field notes made during and/or after the interview or focus group?	5
21. Duration	What was the duration of the interviews or focus group?	5
22. Data saturation	Was data saturation discussed?	5
23. Transcripts returned	Were transcripts returned to participants for comment and/or correction?	5
Domain 3: analysis and findings		
Data analysis		
24. Number of data coders	How many data coders coded the data?	5
25. Description of the coding tree	Did authors provide a description of the coding tree?	7
26. Derivation of themes	Were themes identified in advance or derived from the data?	5
27. Software	What software, if applicable, was used to manage the data?	4
28. Participant checking	Did participants provide feedback on the findings?	4
Reporting		
29. Quotations presented	Were participant quotations presented to illustrate the themes / findings? Was each	8-13

	quotation identified? e.g. participant number	
30. Data and findings consistent	Was there consistency between the data presented and the findings?	8-12
31. Clarity of major themes	Were major themes clearly presented in the findings?	8-17
32. Clarity of minor themes	Is there a description of diverse cases or discussion of minor themes?	8-17
