# Supplementary material for: Facilitators and Barriers to Self-Volume Management in Older Patients with Chronic Heart Failure and Multimorbidity: A Qualitative Study
Source: Healthcare (Basel). 2025 Sep 18;13(18):2353. doi: 10.3390/healthcare13182353 (PMC12469624; doi:10.3390/healthcare13182353)
Supplement: Supplementary file 1 [file healthcare-13-02353-s001.zip › File S2 Interview outline.pdf]

File S2: interview outline

Domain	Questions
**Innovation**	(1) What are your main sources of knowledge related to self-volume management? For example, doctors, nurses, promotional materials, or experiences shared by other patients?<br>(2) How much do you trust these sources of information? Why?<br>(3) Which sources do you prefer to obtain information from? If there were more scientific studies or case studies on the effectiveness of volume management, would you be willing to learn about them? Why?<br>(4) What do you think about the importance of self- volume management?<br>(5) If volume management methods could directly improve your quality of life, would you participate more actively? Why?<br>(6) If the volume management approach could be adapted more flexibly to your lifestyle, would you be more willing to adhere to it in the long term? Why?
**Outer Setting**	(1) To what extent do you think the external environment (healthcare professionals, friends, etc.) supported your volume management implementation? Were adequate resources provided, e.g., educational materials, equipment, etc.?<br>(2) To what extent do you think this support influenced the effectiveness of your volume management implementation?<br>(3) How do you think local conditions (economic, environmental, medical conditions, health care policies, etc.) facilitated or hindered your implementation of volume management?<br>(4) Do you feel pressure from healthcare organizations, such as reminders or urges to implement volume management?<br>(5) Have these pressures been facilitating or burdensome for you?

	(6) Do you feel that social advocacy or community events could help you focus more on volume management? Why? (How could they help?)<br>(7) What types of training and guidance (e.g., online videos, offline training, microblogging, etc.) do you think could support you in volume management? What information and training are you currently missing?
**Inner Setting**	(1) To what extent do you think internal environmental factors (family members, fluid measurement tools, etc.) support your volume management implementation?<br>(2) To what extent do you think these supports have influenced the effectiveness of your volume management implementation?<br>(3) Did you feel pressure from family members, e.g. to remind or urge you to implement volume management? Were these pressures facilitating or burdensome for you?
**Individual**	(1) Volume management requires you to know your volume status, adhere to a low-sodium diet, record fluid intake and output, and follow up regularly; do you think you have this knowledge and these skills? What kind of help do you need to improve your volume management ability?<br>(2) Do you think you have the conditions for volume management (time, physical condition, work constraints, living conditions, etc.)?<br>(3) Are you willing to take the initiative to implement volume management? What would you like us to do to help you improve your motivation?<br>(4) How confident are you in adhering to volume management? What factors have strengthened or weakened your confidence?<br>(5) During the management process, have you encountered situations that made you feel frustrated or want to give up? What

	were the specific situations?<br>(6) If there were incentives (e.g., regular feedback, goal rewards), would they have improved your persistence? Which incentives do you prefer? Why?
**Implementation Process**	(1) Do you find volume management measures complex? How do you set goals, develop plans, and implement these measures in your daily life?<br>(2) What obstacles or challenges have you encountered when practicing volume management? Do you usually follow your doctor's advice and instructions?<br>(3) How much cost/expense do you incur in the process of volume management?<br>(4) How effective do you think your volume management is? In what areas can adjustments be made?
